# Supplementary material for: Safety Profile and Outcomes of Early COVID-19 Treatments in Immunocompromised Patients: A Single-Centre Cohort Study
Source: Biomedicines. 2022 Aug 18;10(8):2002. doi: 10.3390/biomedicines10082002 (PMC9405567; doi:10.3390/biomedicines10082002)
Supplement: Supplementary file 1 [file biomedicines-10-02002-s001.zip › biomedicines-1856682-SM.pdf]

Methods

Italian National Health System indications to administer RMD and mAbs.

Sotrovimab, casirivimab/imdevimab, bamlanivimab/etesevimab

Use is only recommended in patients older than 12 years, with a weight above 40 kg, with mild or moderate COVID-19, not-hospitalized for COVID-19, with an onset of symptoms not exceeding 7 days.

MAbs could be administered more than 7 days after symptom onset in immunecompromised patients with a negative SARS-CoV-2 serology and prolonged nasopharyngeal swab positivity.

Patients were required to have at least one of the following risk factors:

- age of 65 years or older,
- uncontrolled diabetes or diabetes with chronic complications,
- obesity (body mass index >30; calculated as weight in kilograms divided by height in meters squared),
- primary or secondary immunodeficiency,
- chronic kidney disease (estimated glomerular filtration rate <60 mL/min/1.73 m^2 including peritoneal dialysis or haemodialysis),
- cardio-cerebrovascular disease (including hypertension with concomitant organ damage)
- chronic obstructive pulmonary disease or other chronic respiratory disease
- chronic liver disease
- haemoglobinopathies
- neurodevelopmental and neurodegenerative diseases

Patients were excluded if they were hospitalized or if they had signs or symptoms of severe COVID-19 (shortness of breath at rest, oxygen saturation level <94%, or required supplemental oxygen for COVID-19) or if they have had an allergic reaction to components of the drug or other mAbs in the past.

Sotrovimab was administered as a single dose of 500 mg of sotrovimab intravenously.

Casirivimab/imdevimab was administered as a single dose of casirivimab 600 mg and imdevimab 600 mg intravenously.

Bamlanivimab/etesevimab was administered as a single dose of bamlanivimab 700 and etesevimab 1400 mg intravenously.

Remdesivir

Use is only recommended in patients older than 12 years old, with a weight above 40 kg with mild or moderate COVID-19, not-hospitalized for COVID-19, with an onset of symptoms not exceeding 7 days.

Patients were required to have at least one of the following risk factors:

- oncological/oncohaematological pathology in active phase
- uncontrolled diabetes or diabetes with chronic complications,
- obesity (body mass index >30; calculated as weight in kilograms divided by height in meters squared),
- primary or secondary immunodeficiency,
- chronic kidney disease (with an estimated glomerular filtration rate < 60 mL/min/1.73 m^2 but >30 mL/min/1.73 m^2 excluding peritoneal dialysis or haemodialysis),
- severe cardiovascular disease (chronic heart failure, coronary artery disease, cardiomyopathy),
- chronic obstructive pulmonary disease or other chronic respiratory disease
- chronic liver disease

Patients were excluded if they had ALT >5 UL N at the blood tests, an estimated glomerular filtration rate < 30 mL/min/1.73 m^2, if they were hospitalized or if they had signs or symptoms of severe COVID-19 (shortness of breath at rest, oxygen saturation level <94%, or required supplemental oxygen for COVID-19).

Remdesivir was administered at a dosage of 200 mg on day one then 100 mg on day two and three.
