# Supplementary material for: Safety Profile and Outcomes of Early COVID-19 Treatments in Immunocompromised Patients: A Single-Centre Cohort Study
Source: Biomedicines. 2022 Aug 18;10(8):2002. doi: 10.3390/biomedicines10082002 (PMC9405567; doi:10.3390/biomedicines10082002)
Supplement: Supplementary file 1 [file biomedicines-10-02002-s001.zip › Table S1. Co-morbidities by subjectsí» immune status.pdf]

Table S1. Co-morbidities by subjects' immune status.

			Immunocompromised		
Co-morbidities	N	Overall, N = 143*	No, N = 37*	Yes, N = 106*	p-value†
**Myocardial infarction**	143	7 (4.9%)	3 (8.1%)	4 (3.8%)	0.375
**Chronic heart failure**	143	0 (0%)	0 (0%)	0 (0%)	-
**Peripheral vascular disease**	143	7 (4.9%)	3 (8.1%)	4 (3.8%)	0.375
**Stroke or TIA**	143	3 (2.1%)	1 (2.7%)	2 (1.9%)	>0.999
**Dementia**	143	1 (0.7%)	0 (0%)	1 (0.9%)	>0.999
**COPD**	143	5 (3.5%)	1 (2.7%)	4 (3.8%)	>0.999
**Peptic ulcer disease**	143	1 (0.7%)	0 (0%)	1 (0.9%)	>0.999
**Liver disease**	143				0.489
Mild		10 (6.9%)	1 (2.7%)	9 (8.5%)	
Moderate		2 (1.4%)	0 (0%)	2 (1.9%)	
Severe		0 (0%)	0 (0%)	0 (0%)	
**Diabetes mellitus**	143				0.798
Uncomplicated		22 (15%)	5 (14%)	17 (16%)	
End-organ damage		0 (0%)	0 (0%)	0 (0%)	
**Hemiplegia**	143	0 (0%)	0 (0%)	0 (0%)	-
**Moderate to severe chronic kidney disease**	143	37 (26%)	4 (11%)	33 (31%)	0.015
**KDIGO Classification**	107				0.720
Stage 1		44 (41%)	11 (55%)	33 (38%)	
Stage 2		29 (27%)	5 (25%)	24 (28%)	
Stage 3a		15 (14%)	3 (15%)	12 (14%)	
Stage 3b		10 (9.3%)	1 (5.0%)	9 (10%)	
Stage 4		7 (6.5%)	0 (0%)	7 (8.0%)	
Stage 5		2 (1.9%)	0 (0%)	2 (2.3%)	
**Dialysis**	143	3 (2.1%)	0 (0%)	3 (2.8%)	0.569
**Lung disease**	142				0.202
Asthma		4 (2.8%)	3 (8.3%)	1 (0.9%)	
Fibrosis		1 (0.7%)	0 (0%)	1 (0.9%)	
COPD		5 (3.5%)	1 (2.8%)	4 (3.8%)	
Cystic fibrosis		5 (3.5%)	0 (0%)	5 (4.7%)	
Other		10 (7.0%)	3 (8.3%)	7 (6.6%)	

			Immunocompromised		
Co-morbidities	N	Overall, N = 143*	No, N = 37*	Yes, N = 106*	p-value†
**IBD**	143	3 (2.1%)	0 (0%)	3 (2.8%)	0.569
**Neurological disease**	143				0.880
Neurodevelopmental disorder		2 (1.4%)	0 (0%)	2 (1.9%)	
Neurodegenerative disorder		5 (3.5%)	2 (5.4%)	3 (2.8%)	
Other(s)		9 (6.3%)	2 (5.4%)	7 (6.6%)	
**Home oxygen therapy**	143	1 (0.7%)	0 (0%)	1 (0.9%)	>0.999

*Median (IQR) or Frequency (%)

†Fisher's exact test; Pearson's Chi-squared test

Abbreviations: COPD=chronic obstructive pulmonary disease; IBD=intestinal bowel diseases; KDIGO=Kidney Disease Improving Global Outcomes; TIA=transient ischemic attack
