# Supplementary material for: Safety Profile and Outcomes of Early COVID-19 Treatments in Immunocompromised Patients: A Single-Centre Cohort Study
Source: Biomedicines. 2022 Aug 18;10(8):2002. doi: 10.3390/biomedicines10082002 (PMC9405567; doi:10.3390/biomedicines10082002)
Supplement: Supplementary file 1 [file biomedicines-10-02002-s001.zip › Table S2. COVID-19 symptoms at the time of treatment prescription by subjectsí» immune status.pdf]

Table S2. COVID-19 symptoms at the time of treatment prescription by subjects' immune status.

			Immunocompromised		
COVID-19 signs and symptoms	N	Overall, N = 143*	No, N = 37*	Yes, N = 106*	p-value†
**Fever**	143	78 (55%)	16 (43%)	62 (58%)	0.109
**Cough**	143	75 (52%)	25 (68%)	50 (47%)	0.032
**Ageusia-dysgeusia**	143	7 (4.9%)	4 (11%)	3 (2.8%)	0.074
**Anosmia**	143	7 (4.9%)	4 (11%)	3 (2.8%)	0.074
**Pharyngodynia**	143	45 (31%)	8 (22%)	37 (35%)	0.134
**Asthenia**	143	31 (22%)	9 (24%)	22 (21%)	0.650
**Headache**	143	32 (22%)	9 (24%)	23 (22%)	0.741
**Myalgia**	143	22 (15%)	2 (5.4%)	20 (19%)	0.051
**GI signs and symptoms**	143	15 (10%)	6 (16%)	9 (8.4%)	0.216
**Dyspnoea - tachypnoea**	143	5 (3.5%)	3 (8.1%)	2 (1.9%)	0.109

*Median (IQR) or Frequency (%)

†Pearson's Chi-squared test; Fisher's exact test

Abbreviations: GI=gastrointestinal
