# Supplementary material for: Safety Profile and Outcomes of Early COVID-19 Treatments in Immunocompromised Patients: A Single-Centre Cohort Study
Source: Biomedicines. 2022 Aug 18;10(8):2002. doi: 10.3390/biomedicines10082002 (PMC9405567; doi:10.3390/biomedicines10082002)
Supplement: Supplementary file 1 [file biomedicines-10-02002-s001.zip › Table S3. Specific adverse drug reactions events by subjectsí» immune status.pdf]

Table S3. Specific adverse drug reactions events by subjects' immune status.

			Immunocompromised	
Adverse events (*specification*)	N	Overall, N = 143*	No, N = 37*	Yes, N = 106*
**Early ADRs**	143	1 (0.7%)	0 (0%)	1 (0.9%)
**Late ADRs**	143	22 (15%)	6 (16%)	16 (15%)
Skin rash	143	3 (2.1%)	1 (2.7%)	2 (1.9%)
Itching	143	2 (1.4%)	1 (2.7%)	1 (0.9%)
Injection site reactions	143	2 (1.4%)	2 (5.4%)	0 (0%)
Urticaria	143	1 (0.7%)	0 (0%)	1 (0.9%)
Dizziness	143	1 (0.7%)	1 (2.7%)	0 (0%)
Asthenia	143	2 (1.4%)	0 (0%)	2 (1.9%)
Chills	143	1 (0.7%)	0 (0%)	1 (0.9%)
Fever	143	3 (2.1%)	2 (5.4%)	1 (0.9%)
Tachycardia	143	1 (0.7%)	1 (2.7%)	0 (0%)
Vomiting	143	1 (0.7%)	0 (0%)	1 (0.9%)
Diarrhoea	143	1 (0.7%)	0 (0%)	1 (0.9%)
Other(s)	143	4 (2.8%)	0 (0%)	4 (3.8%)

*Median (IQR) or Frequency (%)

Abbreviations: ADRs= adverse drug reactions
