# Supplementary material for: Safety Profile and Outcomes of Early COVID-19 Treatments in Immunocompromised Patients: A Single-Centre Cohort Study
Source: Biomedicines. 2022 Aug 18;10(8):2002. doi: 10.3390/biomedicines10082002 (PMC9405567; doi:10.3390/biomedicines10082002)
Supplement: Supplementary file 1 [file biomedicines-10-02002-s001.zip › Table S5-Timing from treatment to key parameters by subjectsí» immune status..pdf]

Table S5. Timing from treatment to key parameters by subjects' immune status.

			Immunocompromised		
	N	Overall, N = 143*	No, N = 37*	Yes, N = 106*	p-value†
**Time from treatment start to SARS-CoV-2 test negativity**	132	10 (6, 16)	9 (6, 14)	10 (6, 17)	0.549
**Time from treatment start to COVID-19 symptom resolution**	123	2.0 (1.0, 6.0)	3.0 (1.5, 5.0)	2.0 (1.0, 6.2)	0.639

*Median (IQR) or Frequency (%)

†Wilcoxon rank-sum test; Fisher's exact test
