# Supplementary figures and images for: Safety Profile and Outcomes of Early COVID-19 Treatments in Immunocompromised Patients: A Single-Centre Cohort Study
Source: Biomedicines. 2022 Aug 18;10(8):2002. doi: 10.3390/biomedicines10082002 (PMC9405567; doi:10.3390/biomedicines10082002)

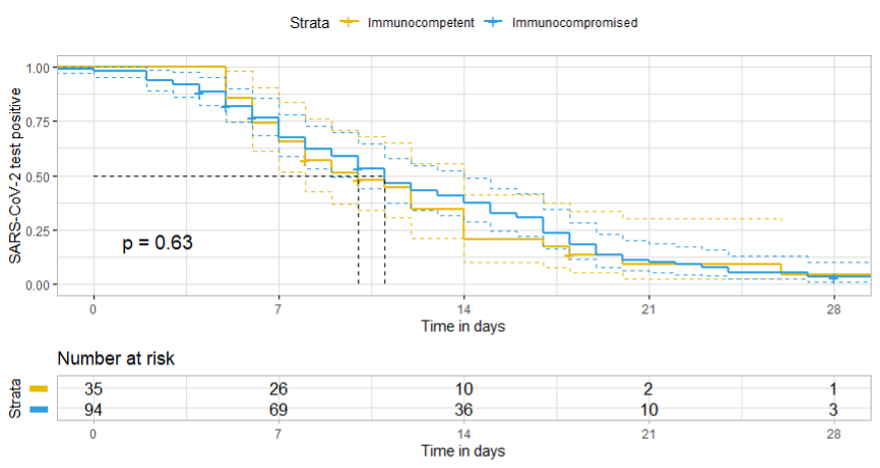

Supplement: Supplementary file 1 [file biomedicines-10-02002-s001.zip › Figure S1. Time from treatment start and SARS-CoV-2 nasopharyngeal swab negativity by subjectí»s immune status.png]

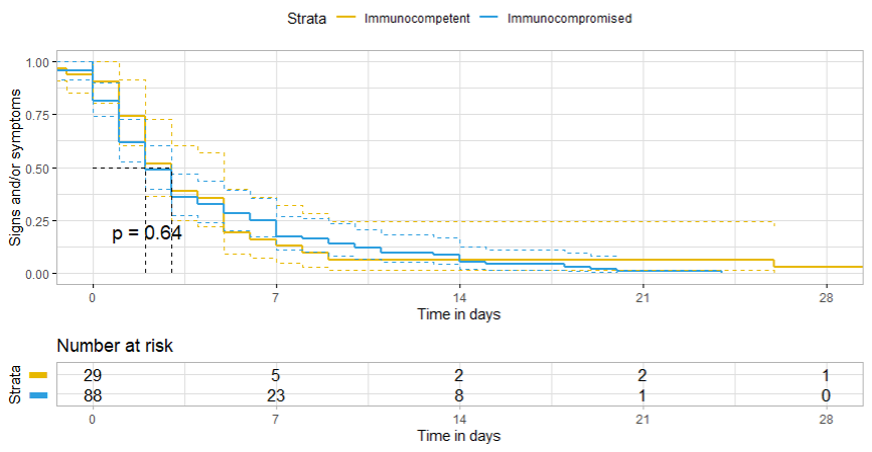

Supplement: Supplementary file 1 [file biomedicines-10-02002-s001.zip › Figure S2. Time from treatment start and COVID-19 signs_symptoms resolution by subjectí»s immune status.png]

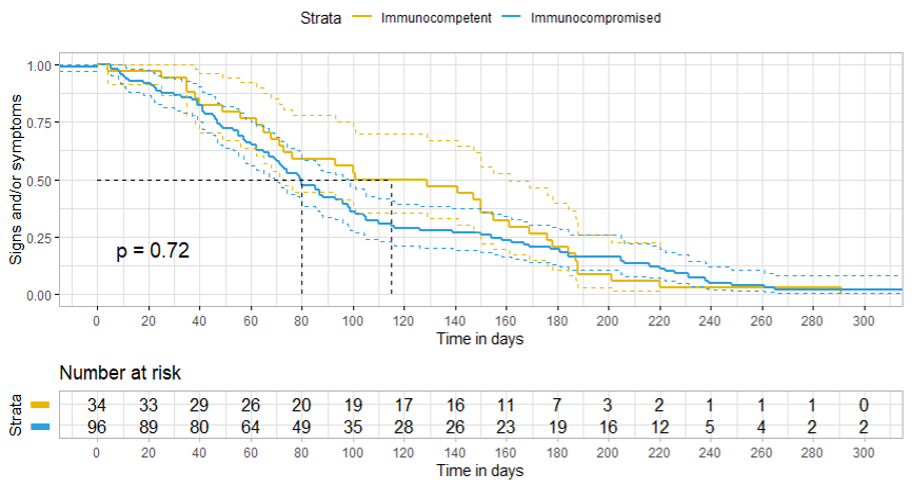

Supplement: Supplementary file 1 [file biomedicines-10-02002-s001.zip › Figure S3. Time from last COVID-19 vaccine dose administration and COVID-19 signs_symptoms onset by subjectí»s immune status.png]
